# An analysis of suspected microplastics in the muscle and gastrointestinal tissues of fish from Sarasota Bay, FL: exposure and implications for apex predators and seafood consumers

**DOI:** 10.3390/environments11090185

**Published:** 2024-08-24

**Authors:** Eric Conger, Miranda Dziobak, Elizabeth J. Berens McCabe, Tita Curtin, Ayushi Gaur, Randall S. Wells, John E. Weinstein, Leslie B. Hart

**Affiliations:** 1 Department of Biology, School of Sciences, Mathematics, and Engineering, College of Charleston, Charleston, SC, USA; 2 Department of Health and Human Performance, School of Health Sciences, College of Charleston, Charleston, SC, USA; 3 Department of Environmental Health Sciences, Arnold School of Public Health, University of South Carolina, Columbia, SC, USA; 4 Sarasota Dolphin Research Program, Brookfield Zoo Chicago, c/o Mote Marine Laboratory, Sarasota, FL, USA; 5 Department of Biology, The Citadel, Charleston, SC, USA

**Keywords:** plastic pollution, bottlenose dolphin, One Health, contaminant, trophic transfer

## Abstract

Microplastics have been found in the gastrointestinal (GI) fluid of bottlenose dolphins (*Tursiops truncatus*), inhabiting Sarasota Bay, FL, suggesting exposure by ingestion, possibly via contaminated fish. To better understand the potential for trophic transfer, muscle and GI tissues from 11 species of dolphin prey fish collected from Sarasota Bay were screened for microplastics (particles <5 mm diameter). Suspected microplastics were found in 82% of muscle samples (*n=89*), and 97% of GI samples (*n=86*). Particle abundance and shapes varied by species (*p<0.05*) and foraging habit (omnivore vs. carnivore, *p<0.05*). Pinfish (*Lagodon rhomboides*) had the highest particle abundance for both tissue types (muscle: 0.38 particles/g; GI: 15.20 particles/g), which has implications for dolphins as they are a common prey item. Findings from this study support research demonstrating the ubiquity of estuarine plastic contamination and underscore the risks of ingestion exposure for wildlife and potentially seafood consumers.

## 1. Introduction

Microplastics are plastic particles less than 5mm in diameter [1,2] and are found everywhere, including terrestrial [3], polar [4], freshwater [5–7], and marine environments [8]. Additionally,[8] estimated that our oceans contain roughly 171 trillion plastic particles. Microplastics enter the environment through various pathways, including degradation of macroplastic litter [9–11], direct contamination as primary microplastics (i.e., microbeads from personal care products; [12–14]), landfill and urban runoff [15–17], or via sewage and wastewater discharge [18,19].

Although widespread, the extent of microplastic contamination is not spatially uniform, and the variability in contamination can be attributed to particle properties (e.g., density and surface area, [20–22]), degrees of urbanization [23–25], and oceanographic currents [26–28]. For instance, particles with a higher density are more likely to sink and accumulate in sediment layers [20,22], and particles with larger surface areas may serve as substrates for biofouling, which can also contribute to their descent in water [21,22]. Geographically, the impact of urbanization on marine microplastic pollution is evident, with studies showing that waters surrounding urban centers are significantly more polluted than those near rural coastlines [24,29], likely due to urban runoff and wastewater discharge [15,17,25]. Additionally, research has demonstrated that microplastic abundance decreases as the distance from shoreline increases, in both rural and urban settings [23,25]. The influence of ocean currents is significant, with lower microplastic concentrations found in regions with strong currents and higher concentrations in areas with slower-moving currents, particularly noticeable in ocean gyres where both micro- and macro-plastics are trapped and circulate indefinitely [26–28].

Although the distribution of marine microplastics varies, their widespread prevalence makes all marine fauna vulnerable to exposure. Microplastics have been detected in multiple tissue types (e.g., muscle, liver, gills, gastrointestinal tracts, “GI”; [30–33]) across a wide range of taxa including jellyfish [34], bivalves [35–37], crustaceans [38], cephalopods [39], turtles [40], marine mammals[41–50], and numerous fish species [30,31,51,52]. Studies indicate that fish harbor the highest concentration of microplastics within their gastrointestinal tracts [53], with fibers being the most commonly observed shape [52,54]. Microplastic exposure in fish can occur via branchial intrusion in which particles enter via gills [32,53,55–57], as well as by incidental or direct ingestion [16,32,34,53,55,56,58–60]. For example, visually-oriented predators can mistake microplastics for prey due to their size and color resemblance [56,59,60], leading to higher gastrointestinal concentrations than chemosensory foragers [56]. However, not all fish actively pursue microplastics or confuse them for food; ingestion can also be an unintentional consequence of feeding in contaminated water [55] or through trophic transfer. An experimental study demonstrated that snowy sculpin (*Myoxocephalus brandti*) had higher concentrations of microplastics in their gastrointestinal tracts when placed in tanks with mysids (*Neomysis* spp) that had previously ingested microplastics, compared to tanks with only suspended microplastics [58]. Trophic transfer has also been observed in higher-order taxa such as grey seals (*Halichoerus grypus*), in which analyses of scat samples revealed microplastic characteristics similar to those found in their prey fish [48].

Recent studies of free-ranging bottlenose dolphins (*Tursiops truncatus*) in Sarasota Bay, Florida, demonstrated prevalent exposure to plasticizers [61,62] and microplastic ingestion [44]. Given previous evidence of microplastics in fish tissues and the potential for trophic transfer, we suspect that dolphin microplastic and plasticizer exposure is likely due to the consumption of contaminated prey. Building on initial findings from a study that compared microplastics in fish tissues and dolphin gastric fluid [51], we sought to quantify and characterize microplastics in a broader and more diverse sample of fish species, which are part of the Sarasota Bay bottlenose dolphin diet. Findings from this study will enhance our understanding of the types of microplastics that fish are exposed to, identify microplastic trophic exposure risks for bottlenose dolphins and local seafood consumers, and support ongoing efforts to monitor microplastic contamination in Sarasota Bay.

## 2. Materials and Methods

### Study Location

**Figure 1 -:**
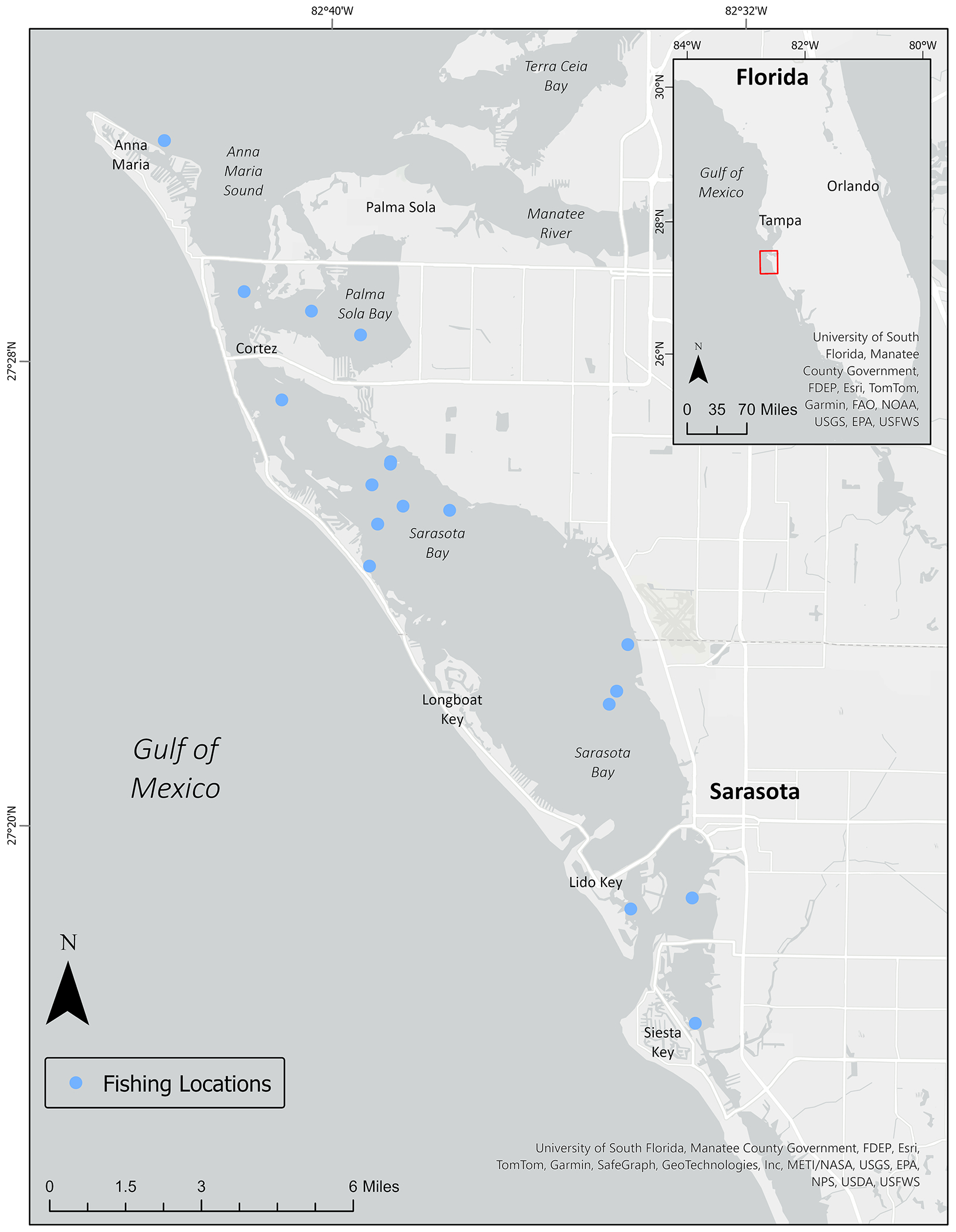
Fish collection locations in Sarasota Bay, Florida, USA (September 2022 - July 2023).

The Sarasota Bay estuary (Figure 1) spans 50 miles along the central Gulf Coast of Florida and connects to the Gulf of Mexico through four inlets or passes [63]. Although tides are shallow (less than 2 feet), tidal exchange with the Gulf of Mexico is the dominant force for water circulation within Sarasota Bay. Several tidal creeks empty into Sarasota Bay along the eastern coast, with drainage areas varying in size (smallest: Palma Sola Creek, 900 acres; largest: Phillippi Creek, 36,417 acres [64]). Daily freshwater inflow averages 11.33 m^3^/s, and salinity throughout the Bay averages 30.00 ppt [65]. Sarasota Bay is an urbanized watershed and consists of agricultural, residential, commercial, and industrial land uses, so stormwater runoff due to the 45 inches of annual rainfall can be a significant contributor of pollutants to the Bay [64]. In fact, nitrogen deposition from wastewater and stormwater is the primary pollution concern for Sarasota Bay, but concentrations have been declining in recent years as a result of changes in stormwater management and efforts to improve wastewater treatment practices [64]. For example, septic systems have been transitioned to centralized sewer systems and regional wastewater treatment plants have been converted to pump stations for transport to larger, centralized facilities. Sarasota County now has three centralized wastewater treatment plants with capacities ranging between 3 and 12 million gallons per day, and reclaimed water is stored in tanks or ponds for residential, municipal and commercial irrigation practices throughout the county [66]. Each plant has plans to become an advanced wastewater treatment (AWT) facility [66], which can be over 90% effective in removing microplastics if employing both primary (i.e., physical process) and secondary (i.e., biological process) treatment practices [67]. In 1989, the United States Congress designated the bay as an estuary of national significance [68], leading to initiatives aimed at reducing pollution, including measures such as plastic straw bans and mandatory recycling protocols [69]. Despite these efforts, recent studies have found evidence of microplastics within the gastrointestinal tracts of fish and dolphins inhabiting the area [44,51].

### Fish Collection

Fish for this study were collected via purse-seining from Sarasota Bay, FL (Figure 1) by the Brookfield Zoo Chicago’s Sarasota Dolphin Research Program (SDRP) between September 2022 and July 2023 as part of efforts to monitor seasonal abundance [70, 71]. SDRP fish survey methods and procedures have been previously described [71,72]. Briefly, the study area for SDRP’s seasonal fish abundance surveys was chosen based on the spatial distribution of the resident dolphins, covering estuarine waters from Passage Key Inlet at the southwestern edge of Tampa Bay (27.5528° N / 82.7423° W) southward to Phillippi Creek, south of Sarasota Bay (27.27096° N / 82.53757° W). Five distinct habitat types were characterized within this study area including creek/mangrove edge, seagrass beds, open bay, sand flat, and nearshore gulf waters, based on location, water depth, and bottom type (vegetated vs. unvegetated) [71]. Using a 200 x 200-m resolution sampling grid originally created in ArcGIS 8.0 (Environmental Systems Research Institute, Redlands, CA, USA), sampling stations were located at the centroids of each grid cell and habitat type was identified at each centroid. Sampling stations were then chosen at random based on habitat type. For the present study, fish were collected during surveys focusing exclusively on seagrass habitat within Sarasota Bay, as the primary prey fish of resident dolphins in Sarasota Bay are associated with seagrass habitat [71]. Twelve species were targeted based on reports from stomach content analyses and observed feeding in the field [72,73]; these included hardhead catfish (*Ariopsis felis*), sheepshead (*Archosargus probatocephalus*), menhaden (*Brevoortia tyrannus*), spotted seatrout (*Cynoscion nebulosus*), ladyfish (*Elops saurus*), scaled sardine (*Harengula jaguana*), pinfish (*Lagodon rhomboides*), spot (*Leiostomus xanthurus*), striped mullet (*Mugil cephalus*), Gulf toadfish (*Opsanus beta*), pigfish (*Orthopristis chrysoptera*), and Atlantic thread herring (*Opisthonema oglinum*). Fish collection was approved by Mote Marine Laboratory’s Institutional Animal Care and Use Committee (IACUC, Permit nos. 22-09-RW2, 23-09-RW2) and Florida Fish and Wildlife Conservation Commission Special Activity License nos. 19-0809A-SR and 22-0809-SR.

### Sample Processing and Analysis

Dissections to remove muscle tissue and the gastrointestinal tract were conducted on metal trays using stainless steel scalpels and forceps, and tissues were stored at −20 °C glass jars until digestion [51]. To digest organic material, muscle and GI tissues were incubated in a potassium hydroxide (KOH, 10%) solution at 60 °C [74] for 24-72 hours. The resulting digestate was vacuum filtered onto a GF/A 1.6 μm glass fiber filter within a fume hood [51,75]. Samples containing large quantities of inorganic solids or durable organic remnants (i.e., sediment, crustacean exoskeletons, bone, and scales) were pre-filtered through 63 μm and 500 μm sieves prior to vacuum filtration. Filters were then placed in covered petri dishes and stored in a cabinet to dry.

Suspected microplastics were visually identified under a Leica EZ4 microscope at 16-35x magnification [74–76]. Characteristics of suspected microplastics included homogenous coloring, absence of organic or cellular structures, and uniform thickness of fibrous particles [74]. Suspected microplastics were categorized by color and shape. Fibers appeared significantly longer than they were wide [2,77], foams were round and porous, changing shape upon touch [2,77–79], films were flat with greater length and width than depth [2,77,79], and fragments had distinct corners [2,77]. Tire wear particles (TWP) were identified as black, rubbery fragments that retained their shape upon manipulation [76]. Plastic testing was conducted with a heated (250 °C) soldering needle [75], which causes plastic particles to bend or melt, as most polymers melt near a temperature of 250°C [74,75,80]. Fourier Transform Infrared (FTIR) spectroscopy (Nicolet iS20, Thermo Scientific, Waltham, MA, USA) was available for polymer determination; however, the particle sizes in this study were smaller than the instrument’s detection threshold (500 μm to 5 mm). Therefore, our findings report suspected microplastics identified via visual characteristics and hot needle responses [44,51].

### QA/QC

Before each dissection, all tools were triple-rinsed with Milli-Q^®^ purified water [2,74,75]. During the dissections, a petri dish containing a glass fiber filter was placed on the benchtop to capture ambient microplastics, serving as a “dissection blank” [2,74]. This blank was processed identically to the fish tissues to control for ambient contamination. Additionally, 100% cotton lab coats dyed orange (an uncommon microplastic color) and clean nitrile gloves were worn during dissection, digestion, filtration, and counting procedures to avoid contamination by personnel [2,74,76]. For quality assurance and control (QA/QC), blanks were collected at each step of the analysis. One lab/procedural blank without any tissue was processed with each digestion batch to account for contamination during sample processing [74]. To evaluate the efficiency of microplastic recovery, three positive controls containing polyethylene films, polystyrene foams, and polyester fibers were included [2]. These controls demonstrated mean recoveries of 60% for films, 83% for foams, and 85% for fibers. Finally, microplastic particles that matched the shape and color of those found in the corresponding blanks were excluded from the total counts in the sample data [2,51,74].

### Statistical Analysis

The proportion of muscle and GI samples with suspected microplastics was determined for all fish combined and by species. Particle counts were categorized by shape and color and summarized for both tissue types across all species sampled. For each tissue type, particle load was quantified as the number of suspected microplastics per gram of tissue [51]. Mean particle load was compared across species using a Kruskal-Wallis test and between foraging habits (i.e., carnivore vs. omnivore) using a Mann-Whitney U test. All statistical analyses were conducted using Statistica software (version 13, Tibco, Inc., Palo Alto, CA, USA), with statistical significance set at ɑ=0.05.

## 3. Results

### 3.1. Sample Characteristics

From September 2022 through July 2023, 11 fish species were collected from 17 locations in Sarasota Bay (Figure 1). In total, 94 fish were screened for suspected microplastics, with 2 to 24 individuals per species (Table 1). Muscle tissue (*n=89*) mass varied between species, with the largest belonging to the ladyfish (*Elops saurus; n=2*; range: 100.20g - 117.30g), and the smallest belonging to the Atlantic thread herring (*Opisthonema oglinum*; *n=4*; range: 0.90g - 9.20g; Table 1). Among GI samples (n=86), the hardhead catfish (*Ariopsis felis*) had the largest tissue mass (*n=6*; range: 11.40g - 48.90), and the scaled sardine (*Harengula jaguana*) had the smallest (*n=8*; range: 0.50g - 5.10g; Table 1). For all fish, the muscle tissue mass was higher than their GI sample counterpart (Table 1).

### 3.2 Microparticles in Muscle Samples

**Figure 2.**
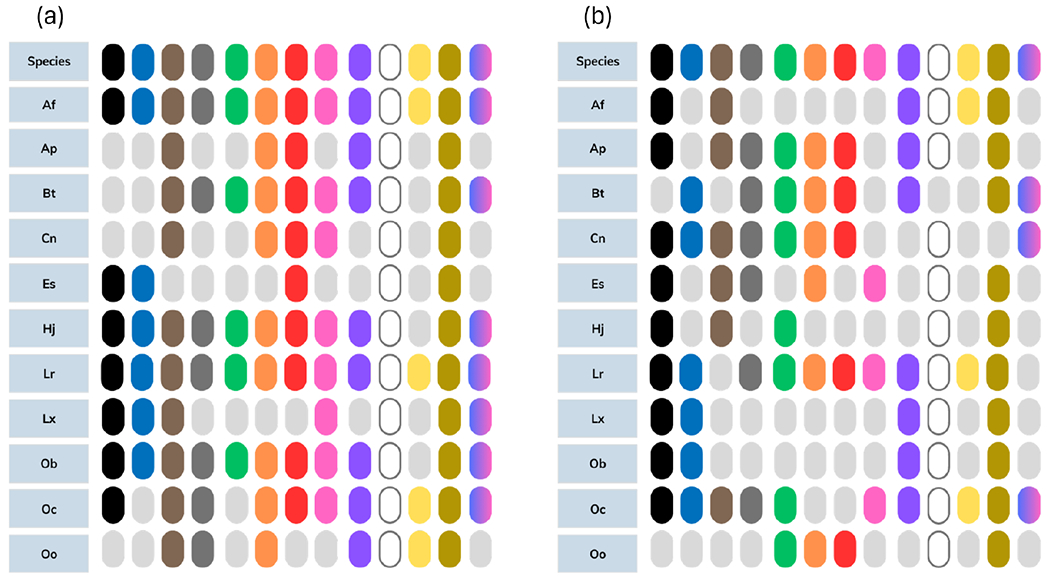
Suspected microplastic colors by species for GI samples (a) and muscle samples (b). From left to right the colors are black, blue, brown, gray, green, orange, red, pink, purple, transparent/white, yellow, yellowed, and multi-colored. From top to bottom the species are Af: hardhead catfish (*Ariopsis felis*); Ap: sheepshead (*Archosargus probatocephalus*); Bt: menhaden (*Brevoortia tyrannus*); Cn: spotted seatrout (*Cynoscion nebulosus*); Es: ladyfish (*Elops saurus*); Hj: scaled sardine (*Harengula jaguana*); Lr: pinfish (*Lagodon rhomboides*); Lx: spot (*Leiostomus xanthurus*); Ob: Gulf toadfish (*Opsanus beta*); Oc: pigfish (*Orthopristis chrysoptera*); and Oo: Atlantic thread herring (*Opisthonema oglinum*).

Suspected microplastic particles were found in 82.02% (*n=73*) of the muscle samples observed. Overall, particle counts in muscle tissue were relatively low; 75.28% of muscle samples contained <10 particles (Table 2). Among the particle shapes observed, single fibers were most common (71.91%), followed by films (26.97%), fragments (11.24%; both non-TWP and TWP), foams (5.62%), and fiber bundles (3.37%; Table 2). No mixed bundles were present. Among the colors observed, yellowed and transparent particles were found in the muscles of every species screened (Figure 2).

### 3.3 Microparticles in Gastrointestinal Samples

Among the 86 GI samples screened, 96.51% (*n=83*) contained at least one suspected microplastic particle. Microparticles were more abundant in GI samples; 60.05% of samples contained 10 particles or more (Table 3). In fact, nearly 300 suspected microplastics were observed in the GI tissue of a single hardhead catfish (Table 3). Particle shapes observed in GI samples varied, but similarly to muscle samples, single fibers were the most common (82.56% of samples screened; Table 3). Films and fiber bundles were also commonly observed (62.79% and 48.84%, respectively), while fewer samples contained fragments (non-TWP: 32.56% and TWP: 16.28%), mixed bundles (12.79%), and foams (4.65%; Table 3). GI particle colors were also variable, but similarly to muscle samples, transparent and yellowed were commonly observed across all species (Figure 2).

### 3.4 Comparisons Across Species

For both muscle and GI samples, the mean particle load (# particles per gram of tissue) was compared across species to account for differences in sample mass. Samples from sheepshead and ladyfish were excluded from species comparisons due to their limited sample size (*n=2*; Table 1). Significant differences in mean particle load were observed across species for both muscle (Kruskal-Wallis, *p=0.006*) and GI tissues (Kruskal-Wallis, *p=0.003*). Additionally, mean particle load was consistently higher for GI samples, compared to muscle tissue (Table 1). Particle abundance was highest in pinfish for both tissue types (*n=24*; muscle: 0.38 particles/g; GI: 15.20 particles/g), and high particle loads were also observed in the Atlantic thread herring (*n=4*; muscle: 1.08 particles/g; GI: 11.61 particles/g).

**Figure 3.**
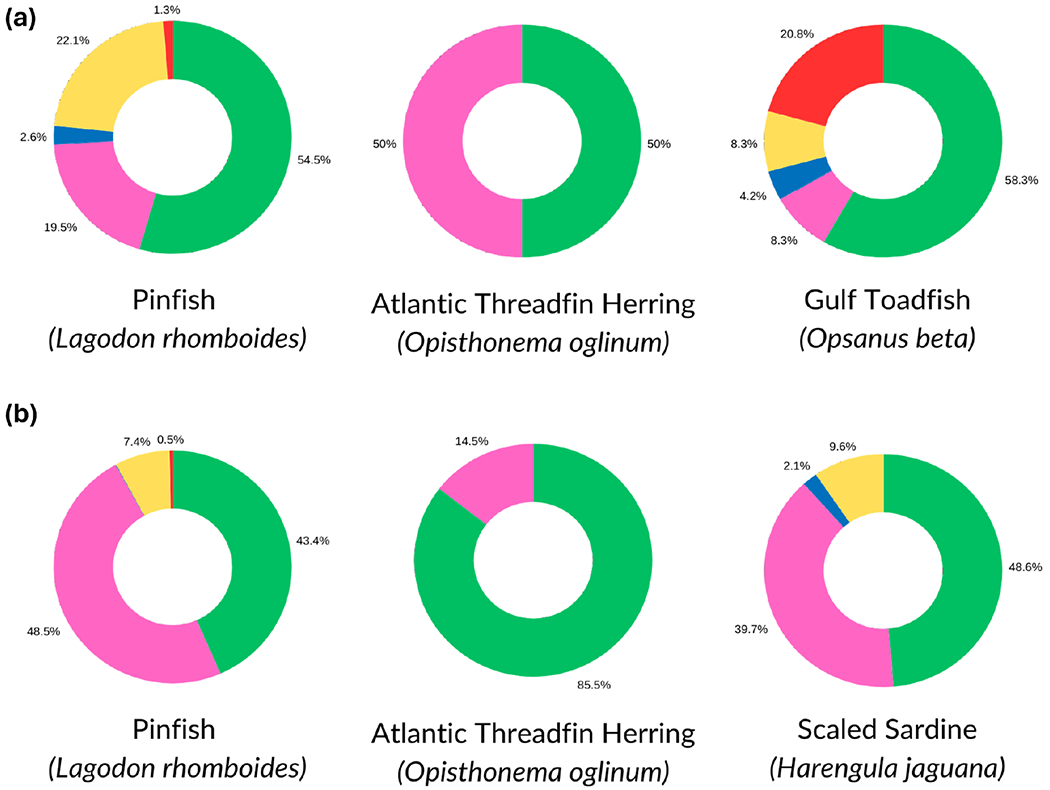
Particle shapes (green = fibers, pink = films, blue = foams, yellow = non-TWP fragments, and red = TWP fragments) among fish species with the highest particle loads for muscle (a) and GI (b) samples.

Among the three species with the highest mean particle load for muscle samples (pinfish, Atlantic threadfin herring, gulf toadfish), fibers were most abundant (Figure 3). Other common particle shapes in muscle samples from these fish included films (pinfish and Atlantic threadfin herring), non-tire wear fragments (pinfish), and tire wear fragments (gulf toadfish; Figure 3). For the fish with the highest particle loads in GI samples (pinfish, Atlantic threadfin herring, scaled sardine), fibers and films were most abundant (Figure 3).

Each fish species was grouped by feeding habit (i.e., carnivore, omnivore, herbivore; [81]; Table 1), for additional comparisons of particle load. Herbivores were not included in the analysis, as none of the species screened were herbivorous. Despite the larger sample size and sample masses for carnivorous fish species (*n = 63*), mean particle load was significantly higher among omnivorous fish (n = 26) for GI samples (14.40 vs. 5.12 particles/g; Mann–-Whitney U test, *p = 0.03*). No significant differences in mean particle load were observed for comparisons of muscle tissue be-tween omnivorous (0.36 particles/g) and carnivorous (0.25 particles/g) fish (Mann–-Whitney U test, *p = 0.11*). It should be noted, however, that the majority of omnivorous fish (92%) were pinfish.

## 4. Discussion

Microplastic contamination of fish commonly consumed by Sarasota Bay dolphins was substantial. Suspected microplastics were observed in 82% of muscle samples (*n = 73*) and 97% (*n = 83*) of GI samples (Tables 2,3), which is higher than some previous studies at other sites. For example, [9] found suspected microplastics in only 35% of GI samples from Nile tilapia (*Oreochromis niloticus*), African sharptooth catfish (*Clarias gariepinus*), common Carp (*Cyprinus carpio*) and Crucian carp (*Carassius carassius*; n=125). Similarly, [82] observed microplastics in approximately 25% of muscle samples and 40% of GI samples from red mullet (*Mullus barbatus*; n= 82) and pontic shad (*Alosa immaculata*; n= 82). Other studies have provided results in a similar range to ours; [53] observed microplastics in 100% of sin croaker (*Johnius dussumieri*) GI samples (n = 188) from Mumbai, India, and, [83] found microplastics in 100% of both GI and muscle samples screened from painted combers (*Serranus scriba*) sampled near the Tunisian coast. The high proportion of fish with suspected microplastics in our study could be attributed to Sarasota Bay’s location. To our knowledge, systematic studies of microplastic pollution in Sarasota Bay have not been performed; however, research by [84] suggested that the neighboring Tampa Bay could contain up to 4 billion microplastic particles. Also, Sarasota Bay is an urban estuary that receives freshwater input from multiple sources. Since freshwater tributaries can carry agricultural and urban runoff, [85] suggest that they may serve as substantial conduits for estuarine microplastics. Additionally, these freshwater creeks can create mixing zones with saltwater from the ocean, potentially trapping debris and acting as a sink for microplastics [86].

Consistent with previous studies [57,82,87], particle counts were higher in GI samples than in muscle tissue, as ingestion is a primary exposure route for microplastics [16,32,34,53,55,56,58–60]. Studies of fish from Turkey, Iran, and Tunisia have shown similar results, with lower concentrations in muscle tissues compared to GI and stomach samples [82,87,88]. The mechanism by which particles enter muscle tissue remains unclear, but it is theorized that they escape the GI tract through cellular gaps in the stomach lining [89]. This translocation hypothesis was demonstrated in European sea bass (*Dicentrarchus labrax*) fed fluorescently-labeled particles (1-5μm; [89]).

Despite all fish being collected within Sarasota Bay (Figure 1), particle abundance varied across species. These findings are consistent with research by the authors of [9], which demonstrated variable contamination in fish sampled from different sites within the same body of water. Differences in foraging habits could help explain this variation by species. For example, studies have shown that benthic fish (such as catfish) ingest more plastic than surface feeders [9,90], likely due to higher concentrations of microplastics in sediment compared to surface waters [20–22]. Additionally, as microplastics undergo weathering and biofouling, they can sink [20–22], increasing the likelihood of consumption by fish that feed lower in the water column.

Our findings suggest that diet may influence contamination, as particle load was higher among omnivorous fish. However, caution is warranted in interpreting this result because the majority of omnivorous fish in our study were pinfish, which had the highest particle abundance. Although pinfish are considered omnivorous, seagrasses, a significant component of their diet [91], could be a substantial sink for microplastics. For example, [92] observed microplastic particles on 75% of examined seagrass blades. Similar trends of higher particle counts in omnivorous fish have been reported in other studies [59,90], where the authors hypothesize that the diverse diet of omnivorous fish increases their chances of ingesting particles. Microplastics have been detected in plants, various fish species, and lower trophic organisms [30,31,92,93], all of which could be food sources for omnivorous fish. Lastly, while some studies suggest that fish may inadvertently consume microplastics that resemble their typical food in color or shape [56,59,60], our results do not support this theory, as we did not observe color preferences among different species.

### Significance of Findings

Our previous studies observed ingested microplastics in Sarasota Bay dolphins [44], and the results of this study provide insight into possible sources of their exposure. We detected suspected microplastics in every fish species examined, all of which are commonly consumed by bottlenose dolphins in Sarasota Bay, Florida, with pinfish being the species most frequently found in Sarasota dolphin stomach contents [72,73,94]. Considering the evidence of trophic transfer in marine mammal studies [48,95], it is possible that contaminated fish could be a substantial source of microplastic exposure for Sarasota Bay dolphins. Although the impacts of microplastic exposure are not yet understood for dolphins and other marine mammals, *in vitro* laboratory studies suggest that adverse health effects such as inflammation [96,97], reproductive impairment [98,99], neurological impairment [100,101], and metabolic issues [102,103] are possible.

Additionally, our findings of microplastics in these fish are concerning for seafood safety. In 2021, Florida was ranked 11th in the United States for the highest production of fresh seafood, accounting for 4.2% of the national total value [104]. The species examined in our study hold commercial value or are sought after for sport fishing [105]. The most contaminated species in our study, the pinfish, is commonly used as bait fish in both commercial and recreational fishing [106,107]. Through trophic transfer, these larger commercial species, such as spotted seatrout, could become contaminated, thereby increasing exposure risk for seafood consumers [108].

### Strengths and Limitations

One challenge for microplastic research is the potential contamination from ambient particles [109]. To mitigate and monitor ambient contamination, several precautions were implemented, such as wearing 100% cotton laboratory coats, rinsing instruments with filtered water, and collecting laboratory and procedural blanks. Additionally, a conservative approach to blank-correction was adopted in which sample particles resembling the shape and color of suspected microplastics in blanks were excluded from abundance counts and particle characterization. This method enhanced the reliability of findings by reducing the likelihood of reporting ambient or procedural contaminants as suspected tissue particles. Particle size was also a limitation of this study, as suspected microplastics were too small to confirm their polymer composition using FTIR, which requires particles to be between 500 μm and 5 mm in diameter. Due to this constraint, particles suspected to be microplastics were identified using microscopy and the hot needle method. The hot needle test is less reliable than FTIR analysis because it depends on specific reactions in plastic that can vary (e.g., burning, melting, curling; [110]). Although the hot needle test is not as precise as FTIR, it can still effectively identify microplastics when used by individuals familiar with plastic reactions to heat [110].

## 5. Conclusion

Plastic pollution is a persistent and widespread issue, leading to ubiquitous microplastic contamination. In this study, we examined the muscle and GI tissues of 11 fish species and found suspected microplastics in each one. These fish are commonly consumed by bottlenose dolphins in Sarasota Bay, Florida, suggesting a trophic exposure route for dolphins and other apex predators. Some species examined are also commonly used as bait fish for commercial fishers, suggesting a risk to seafood safety. However, we detected the fewest particles in fillet tissue, indicating a lower exposure risk compared to apex predators that consume whole fish. Additionally, particle loads were higher in omnivorous fish compared to carnivorous fish, possibly due to their varied diet. Therefore, microplastic exposure through trophic transfer could be higher for apex predators and seafood consumers that eat omnivorous fish. While suspected microplastics are abundant in many of these fish, their small sizes may limit plastic confirmation by standard methodologies (e.g., FTIR). Future fish studies should employ methods that use smaller size thresholds (e.g., micro-Raman spectroscopy).

## Figures and Tables

**Table 1.** Characteristics of fish screened for suspected microplastics. Characteristics include species, foraging type^81^, tissue sample counts, tissue sample mass (g), and mean particle load (# particles / g tissue) for muscle and gastrointestinal (GI) samples.

Common Name (*Genus species*)	Foraging Type^1^	Muscle Samples (n)	Muscle Mass (g) mean (sd)	Muscle Particle Load (# particles/g) mean (sd)	GI Samples (n)	GI Mass (g) mean (sd)	GI Particle Load (# particles/g) mean (sd)
Hard-head Catfish (*Ariopsis felis*)	Carnivore	6	34.33 (14.45)	0.08 (0.06)	6	30.43 (16.69)	6.04 (4.67)
Sheeps-head (*Archosargus probatocephalus*)	Omnivore	2	(16.40 - 47.40)*	(0.06 - 0.27)*	2	(3.10 - 19.90)*	(1.81 - 7.10)*
Menhaden (*Brevoortia tyrannus*)	Carnivore	5	30.18 (11.90)	0.11 (0.07)	4	12.30 (2.99)	2.70 (1.23)
Spotted Seatrout (*Cynoscion nebulosus*)	Carnivore	5	68.66 (78.83)	0.02 (0.05)	5	15.68 (11.81)	0.99 (1.29)
Ladyfish (*Elops saurus*)	Carnivore	2	(100.20 - 117.30)*	(0.04 - 0.15)*	2	(20.60 - 25.00)*	(0.68 - 1.16)*
Scaled Sardine (*Harengula jaguana*)	Carnivore	8	6.46 (2.03)	0.15 (0.12)	8	2.39 (1.49)	10.87 (5.51)
Pinfish (*Lagodon rhomboides*)	Omnivore	24	15.01 (15.02)	0.38 (0.64)	25	4.50 (1.78)	15.20 (22.79)
Spot (*Leiostomus xanthurus*)	Carnivore	5	26.24 (2.83)	0.07 (0.05)	4	5.13 (0.86)	0.91 (0.97)
Gulf Toadfish (*Opsanus beta* )	Carnivore	12	7.06 (3.75)	0.38 (0.56)	12	5.61 (2.86)	4.66 (5.14)
Pigfish (*Orthopristis chrysoptera*)	Carnivore	16	10.19 (7.19)	0.23 (0.18)	15	4.16 (3.76)	4.45 (5.45)
Atlantic Thread Herring (*Opisthonema oglinum*)	Carnivore	4	4.3 (3.55)	1.08 (0.92)	3	3.63 (1.06)	11.61 (11.08)

* Minimum and maximum are presented

**Table 2.** Suspected microplastic abundance in muscle tissue of fish collected from Sarasota Bay, FL (n=89).

Particle Shape	Total Muscle Samples with Particle Shape n (%)	Particle Shapes in Muscle Samples with <10 Particles n (%)	Particle Shapes in Muscle Samples with 10-50 Particles n (%)
Fiber Bundles	3 (3.37)	3 (3.37)	0
Single Fibers	64 (71.91)	63 (70.79)	1 (1.12)
Films	24 (26.97)	24 (26.97)	0
Foams	5 (5.62)	5 (5.62)	0
Non-TWP Fragments	10 (11.24)	9 (10.11)	1 (1.12)
TWP Fragments*	10 (11.24)	10 (11.24)	0

* TWP (tire-wear particle)

**Table 3.** Suspected microplastic abundance in GI tissue of fish collected from Sarasota Bay, FL (n=86).

Particle Shapes Observed In GI Tissue	Total GI Samples with Particle Shape n (%)	Particle Shapes in GI Samples with <10 Particles n (%)	Particle Shapes in GI Samples with 10-50 Particles n (%)	Particle Shapes in GI Samples with 51-100 Particles n (%)	Particle Shapes in GI Samples with 101-300 Particles n (%)
Fiber Bundles	42 (48.84)	26 (30.23)	15 (17.44)	0	1 (1.16)
Single Fibers	71 (82.56)	40 (46.51)	25 (29.07)	2 (2.33)	4 (4.65)
Film	54 (62.79)	40 (46.51)	11 (12.79)	0	3 (3.49)
Foam	4 (4.65)	4 (4.65)	0	0	0
Non-TWP Fragment	28 (32.56)	25 (29.07)	2 (2.33)	1 (1.16)	0
TWP Fragment*	14 (16.28)	14 (16.28)	0	0	0
Mixed Bundle	11 (12.79)	6 (6.98)	5 (5.81)	0	0

* TWP (tire-wear particle)

## Data Availability

Data supporting the reported results can be found in the DRYAD data repository (datadryad.org) using this DOI: 10.5061/dryad.nvx0k6
